# The clinical and radiological effectiveness of autologous bone marrow derived osteoblasts (ABMDO) in the management of avascular necrosis of femoral head (ANFH) in sickle cell disease (SCD)

**DOI:** 10.1186/s40634-022-00449-z

**Published:** 2022-02-17

**Authors:** Mir Sadat-Ali, Abdallah S. Al-Omran, Khalid AlTabash, Sadananda Acharya, Tarek M. Hegazi, Mona I. Al Muhaish

**Affiliations:** 1 Department of Orthopaedic Surgery, College of Medicine, Imam AbdulRahman Bin Faisal University Dammam and King Fahd Hospital of the University, POBOX 40071, AlKhobar, 31952 Saudi Arabia; 2 Stem Cell Unit, College of Public Health, Imam AbdulRahman Bin Faisal University Dammam and King Fahd Hospital of the University, AlKhobar, Saudi Arabia; 3 Department of Radiology College of Medicine, Imam AbdulRahman Bin Faisal University, Dammam, Saudi Arabia

**Keywords:** Stem cell, Osteoblasts, Avascular necrosis of femoral head, Osteonecrosis; sickle cell disease

## Abstract

**Purpose:**

Avascular necrosis of the femoral head is a common issue faced by orthopaedic surgeons that ranges between 10 and 18%, but in patients with SCD, the incidence reaches 30%. There is no definite treatment except joint arthroplasty. Regenerative medicine is an option to cure or delay joint arthroplasty. We report here our experience with the injection of ABMDO to manage ANFH and report our medium-term results, the progression of the ANFH if any and the delay in total hip arthroplasty. (THA).

**Methods:**

Sixty-Three (63) patients with SCD and ANFH were examined and thoroughly investigated, and those who had ANFH < grade II were consented to receive ABMDO. Patients were clinically assessed preoperatively using the Visual analogue scale (VAS), Modified Harris Hips Score (MHHS) and Azam-Sadat Score (ASS) for Quality of Life Score for Chronic Hip Disease. Ten millilitres of bone marrow were aspirated under local anaesthesia and placed in 20 CC of culture media. Osteoblasts were cultured from the aspirated bone marrow. Under anaesthesia, the osteonecrosed lesion was drilled using a 3-mm cannulated drill, and 5 million osteoblasts were injected at the lesion site. Patients were evaluated in the outpatient clinic after 2 weeks. At 4 months, a repeat MRI was done, and patients were followed for a minimum of 2 years.

**Results:**

The average age of patients was 25.93 ± 5.48 years. There were 41 (65%) females and 22 (35%) males. The mean hemoglobin S was 83.2 ± 5.1%. The average follow-up was 49.05 ± 12.9 (range: 24–60) months. TheVAS significantly improved from 7.79 ± 1.06 initially to 4.07 ± 1.08 (*p* < 0.0001) at 2 weeks and continued to improve for the next 24 months, when it was 2.38 ± 0.55 (*p* < 0.0001). The MHHS improved from 41.77 ± 5.37 initially to 73.19 ± 6.48 at 4 months (*p* < 0.001), and at 24 months, it was 88.93 ± 3.6 (*p* < 0.001). The ASS also significantly improved from 2.76 ± 0.49 preoperatively to 7.92 ± 0.09 (*p* < 0.0001) at 24 months. A comparison of the MRI’s from before and after the osteoblast implantation revealed new bone formation and amelioration of the avascular lesions. Three patients were unsatisfied with their outcomes. and one patient suffered a repeat attack of the vaso-occlusive crisis within 6 months of the osteoblast injection.

**Conclusions:**

The results give credence to our earlier short follow-up results showing that osteoblast transplantation has great potential in the healing of avascular lesions. Our study fits the criteria of a Phase II clinical trial, and we believe a larger study equivalent to Phase III numbers should be conducted and include patients with not only SCD but also steroid-induced and idiopathic avascular necrosis.

**Level of evidence:**

II

## Introduction

ANFH, as the name suggests, is the insult to the head of femur which results in the interruption of the blood supply, leading to necrosis. Many diseases and drugs like steroids have been suggested to cause ANFH. In general, the true incidence of ANFH is not known, but in the US, it is reported that up to 600,000 people are affected and another 20,000 new cases are diagnosed every year [1–3]. The figures coming out from China are startling: 8.12 million cases of non-traumatic ANFH annually [4]. In Middle Eastern countries, the majority of ANFH occurs in patients with hemoglobinopathies like SCD [5]. The reported incidence of ANFH in SCD patients in Saudi Arabia is in the range of 25–30%, and many of the patients affected are in their late teens [5]. Many treatments like core decompression [6–9], core decompression and bone grafts [10], vascularized bone grafts [11], extracorporeal shock therapy [12], and bisphosphonate therapy to delay the collapse of the head of the femur [13] have been tried in past decades to relieve symptoms of pain and increase joint mobility.

Since many of the procedures lived up to expectations, clinicians were on the lookout for alternative methods. Bone marrow-derived stem cells appeared to be a good option for regenerating the avascular head. The meta-analysis of Li et al. (2014) showed that such a treatment was feasible and effective [14]. An earlier study published from our institution in which osteoblasts were used had excellent short-term follow-up [15]; hence, we continued to use differentiated osteoblasts in this study as well.

The aim of this work is to report the medium-term results of the clinical treatment of ANFH using ABMDO.

## Patients and Methods

The Institutional Review Board of Imam AbdulRahman Bin Faisal University, Dammam, and Saudi Arabia gave approval for the present study (01–314-2015). During the study period, 116 SCD patients with ANFH were seen, and patients who had total collapse of the head of the femur were excluded from the study. Sixty-three (63) patients with SCD with osteonecrosis of the head of the femur were examined and thoroughly investigated. The blood tests performed were a sickle cell test, hemoglobin electrophoresis, renal function tests, and magnetic resonance imaging (MRI), and those who had ANFH < grade II were consented to receive ABMDO. Patients were clinically assessed preoperatively using MHHS and ASS. Ten CC of bone marrow were aspirated under local anaesthesia close to the anterior superior iliac spine of the iliac crest and placed in 20 CC of culture media. Osteoblasts were cultured from the aspirated bone marrow, and after 21 days, 5 million osteoblasts were ready for injection. The procedure for the culture of the osteoblasts is described in our earlier study with a smaller number of patients, which is standard internationally accepted technique [15].

When the ABMDO were ready for injection, patients were readmitted to the hospital within 4 weeks. As per the hospital and anaesthesia protocol, all patients undergoing general anaesthesia required a hemoglobin S of less than 50% to avoid complications. Hence, all patients had exchange transfusions to bring their hemoglobin S lower than 50% before the injection of osteoblasts.

### Surgical Procedure

Under anaesthesia, patients were placed in the lateral position with the affected hip up. Two guide wires were passed into the area of maximum avascular area. The acceptable wire was over drilled with a 3-mm cannulated drill, the guide wire was removed and the cannulated drill was withdrawn to the beginning of the neck. The drilled portion was washed with normal saline using a long catheter. The drill was used to suck out any remaining saline. Using a long catheter, 5 million osteoblasts were transplanted slowly. The cannulated drill was further withdrawn, and an addition 0.5 ml of saline was pushed through the drill. After 4–5 min, the cannulated drill was removed. The drill site on the skin was closed using 3/0 nylon. Patients were discharged the same evening. Patients were evaluated in an outpatient clinic after 2 weeks. Patients were regularly followed in the outpatient clinic, and at 4 months, patients were assessed for the Azam-Sadat Score (ASS) [16] for Quality of Life Score for Chronic Hip Disease and the MHHS. Patients were clinically evaluated every 6 months. An MRI was done on both hips at 4 months and at the last follow-up if symptoms did not improve. Two musculoskeletal radiologists reviewed the MRI independently and reported the films.

### Statistical Analysis

The data were entered into a database and analysed using the Statistical Package for Social Sciences software, version 23.0 (SPSS Inc., Chicago, IL, USA). The data were presented as a mean ± standard deviation (SD). The mean values with 95% confidence intervals (CI) for each assay’s results were calculated, and a *p*-value < 0.05 was considered significant.

## Results

The average age was 25.93 ± 5.48 years (range: 19–42 years). There were 41 (65%) females and 22 (35%) males. The mean hemoglobin S was 83.2 ± 5.1%. Table 1 gives the demographic data of 63 patients. The average follow-up was 40.05 ± 8.9 (range: 24–48) months. TheVAS significantly improved from an initial 7.79 ± 1.06 before the osteoblast injection to 4.05 ± 1.08 (*p* < 0.001) at 2 weeks. At 24 months, the VAS was 2.38 ± 0.55 (*p* < 0.0001). The MHHS improved from 41.77 ± 5.37initially to 73.19 ± 6.48 at 4 months (*p* < 0.001), and at 24 months, it was 88.93 ± 3.6 (*p* < 0.001). The ASS also significantly improved from 2.76 ± 0.49 preoperatively to 7.92 ± 0.09 (*p* < 0.0001) at 24 months. Table 2 shows the three parameters assessed preoperatively and at 2 weeks, 4 months and 24 months.

**Table 1:** Demographic data of all patients

Age (Years)	25.93 ± 5.48
Females	41
Males	22
Ficat I	5
Ficat II	47
Ficat III	11
Follow Up (Months)	40.05 ± 8.9 (range 24–48)

**Table 2:** Assessment of Pre and Post ABMDO Transplantation

Parameter	Pre operative	Post operative (Weeks)			*P* Value between pre operative and at 104 weeks
		2	16	104	
VAS	7.79 ± 1.06	4.07 ± 1.08	3.38 ± 0.72	2.38 ± 0.55	*P* = 0.001
MHHS	41.77 ± 5.37	49.9 ± 4.92	73.19 ± 6.48	84.8 ± 5.22	*P* = 0.001
ASS	2.76 ± 0.49	4.85 ± 0.87	6.2 ± 0.59	7.92 ± 0.90	*P* = 0.001

*VAS *Visual Analogue Scale, *MHHS * Modified Harris Hip Score, *ASS * Azam-Sadat Score

**Fig. 1:**
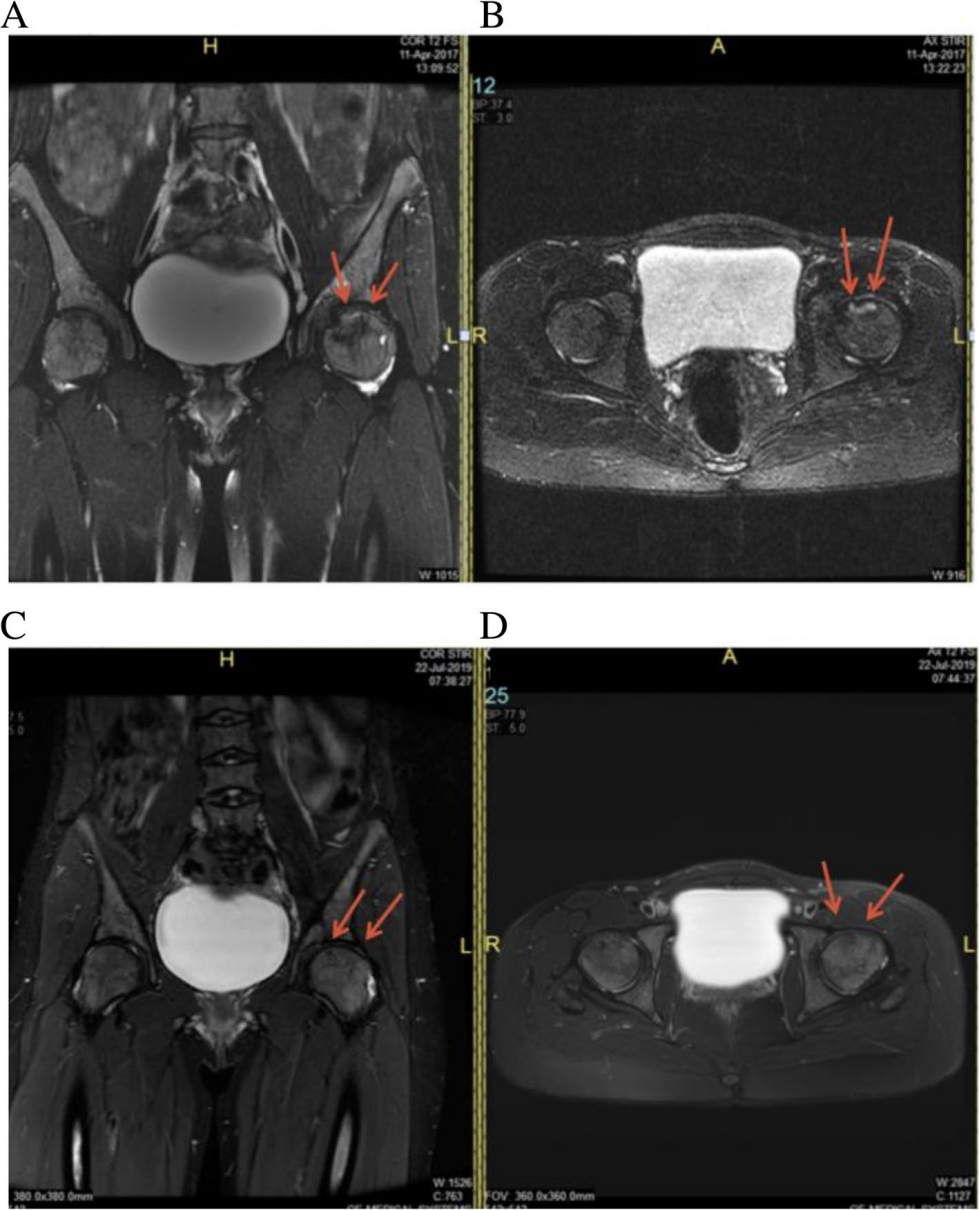
**A** Coronal Short-TI Inversion Recovery image and **B** Axial T2 weighted fat suppressed image of the left hip demonstrates a geographic subchondral area of ANFH in the anterior-superior aspect of the left femoral head. There is no significant subchondral collapse of the femoral head articular surface. **C** Coronal Short-TI Inversion Recovery image and **D** Axial T2 weighted fat suppressed image of the left hip performed 2 years after demonstrates near complete resolution of the femoral head AVN

**Fig. 2:**
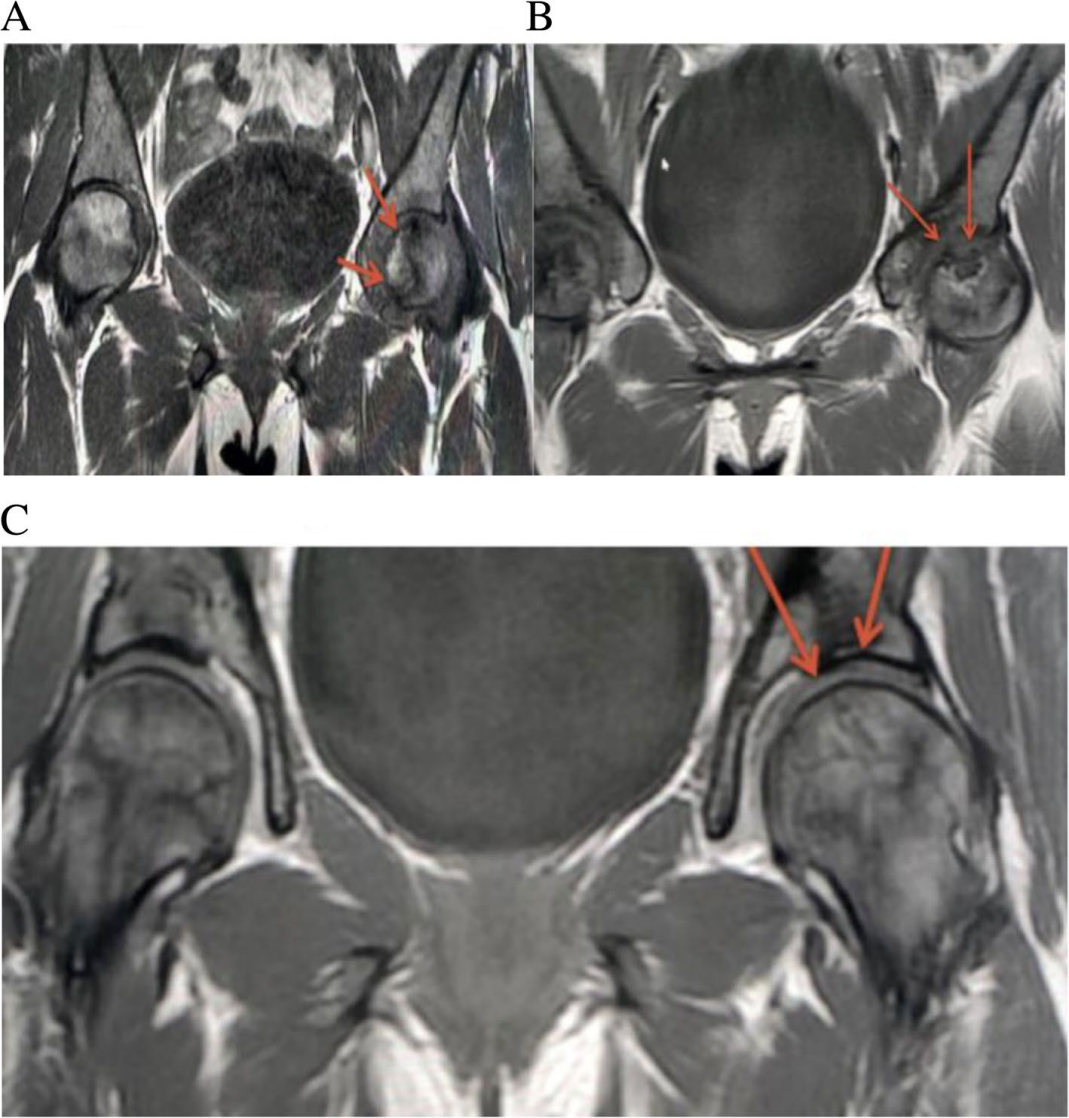
**A** Coronal T1 weighted image of the pelvis demonstrates a large geographic area of ANFH in the anterior-superior aspect of the left femoral head . There is no significant subchondral collapse. **B** Coronal T1 weighted image perfomed 4 months after demonstrates interval improvement of femoral head ANFH. **C** MRI done after 30 months. Coronal Short-TI Inversion Recovery image demonstrates near complete resolution of the femoral head ANFH

**Fig. 3:**
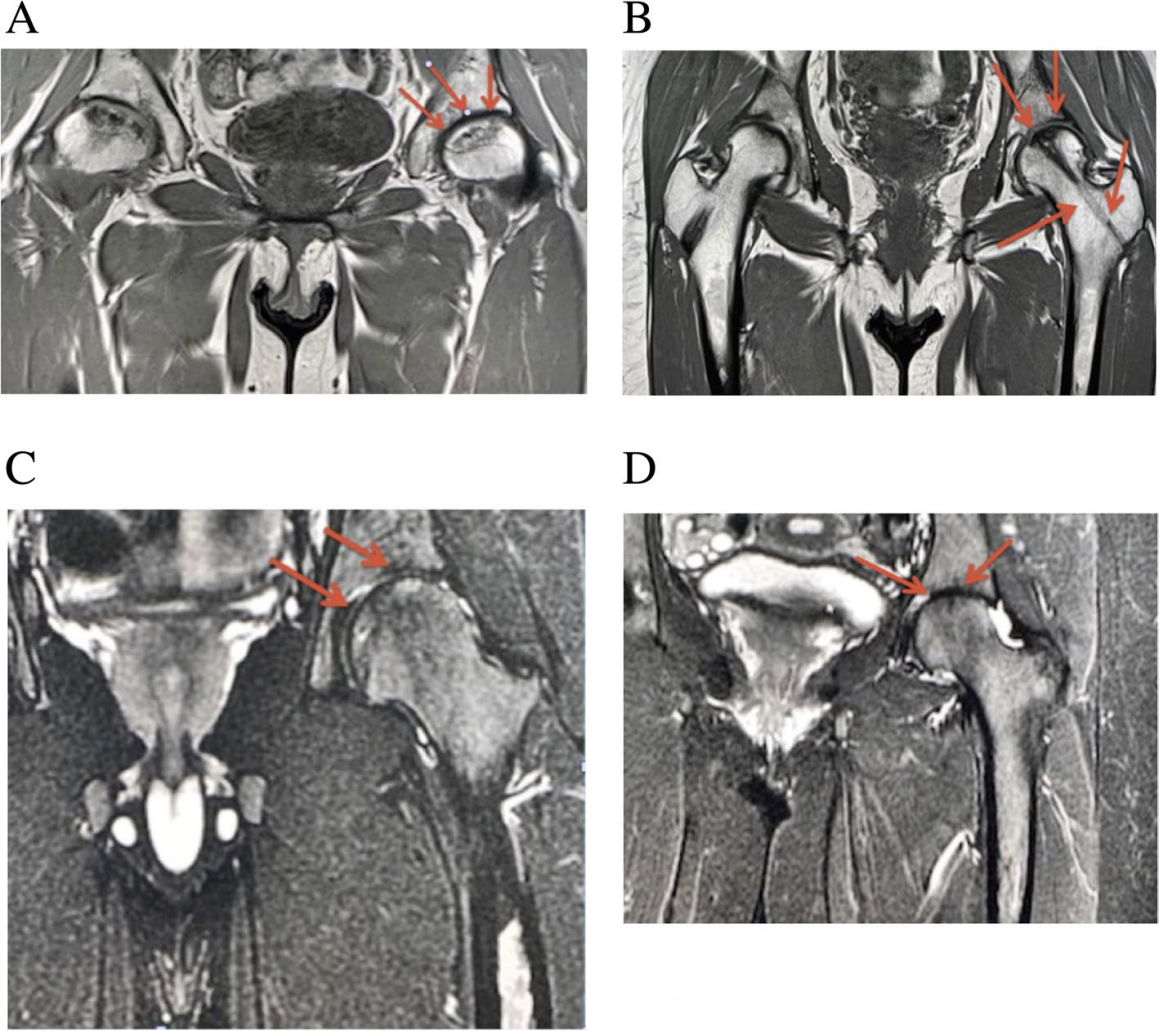
**A** Coronal T1 fat suppressed image of the pelvis demonstrates a geographic area of abnormal signal intensity in the left femoral head (red arrows) compatible with ANFH. **B** Coronal T1 weighted image performed 4 Months after demonstrates interval improvement of ANFH as well ghost tracts from prior drilling through which osteoblasts were transplanted (red arrows). **C** Coronal Short-TI Inversion Recovery weighted image performed 18 Months after demonstrates further interval improvement in surface area involvement of the left femoral head involvement by ANFH (red arrows). **D** Coronal Short-TI Inversion Recovery weighted image performed 36 Months after demonstrates complete resolution of the femoral head ANFH (red arrows)

Overall, 59 (93.6%) patients were satisfied with their improved quality of life, and 4 (6.4%) were unsatisfied, as their disease progressed and they had to have THA (Table 3). The failure of the 4 patients could be due to the occlusion of the vascular supply to the head of the femur due to a vaso-occlusive crisis, and the patients had a hemoglobin S of 92% and Ficat grade III. A comparison of the MRI’s from before and after the osteoblast implantation revealed new bone formation and amelioration of the avascular lesions (Figs. 1, 2 and 3).

**Table 3:** Overall Satisfaction of All Patients

Level of satisfaction	No.(%)
Extremely Satisfied	42 (66.6)
Very Satisfied	11 (17.5)
Satisfied	6 (9.5)
Not Satisfied	4 (6.4%)

## Discussion

Our medium-term results indicate that osteoblasts have the potential to reverse the avascular lesions in the head of the femur in patients with SCD. In addition, the majority of patients were satisfied, and 4 (6.4%) patients underwent THA due to progression of the disease. Their failure could be due to the progression of the disease due to the re-insult of the head of femur from a vaso-occlusive crisis. At the last visit, the remaining patients were not progressing.

Hernigou and Beaujean (2002) [17] treated patients with bone marrow concentrate injections with standard core decompression and follow-up of 60 months and reported that in 6.2% (9 of 145 hips), the disease progressed and the patients required joint replacement. We believe that patients with SCD who have repeated vaso-occlusive crises may end up in failure and may need repeat injections. Kang and his colleagues (2018) [18] performed a comparative study of core decompression and bone marrow mesenchymal stem cell implantation and found that 20% of patients in the stem cell group progressed to clinical failure, while 50% of the hip patients with only core decompression progressed to clinical failure. These reports indicate the bone marrow-derived osteoblasts/mesenchymal stem cells could reverse the avascular lesions; improve the MHHS, VAS and quality of life in the majority of patients; and delay THA. Recently, Palekar et al. (2021) [19] concluded that using osteoblasts in their patients stopped the progression of osteonecrosis and eliminated the need for THA in 70% of their patients.

There is general concern about the safety of stem cell therapies and their side effects. Most of the concerns are related to allogenic stem cells, which can cause tumours and heterotopic ossification [20–22]. However, as it redduced the probability of side effects, autologous stem cell therapy is safer than allogeneic stem cell transplants. This has been proved in longer follow-up studies [23, 24].

This study has limitations. First, there was no control group and no comparison between the conventional core decompression and ABMDO patients, and second, we did not reach the minimum requirement for Phase II of the United States Food and Drug Authority. However, the results of our study, which was prospective nature in and had a medium follow-up, were good. Lastly, in this study, we used subjective and objective assessments, which indicated improved results. We were apprehensive of our results because of the SCD, which can cause re-insults of the hip due to repeated vaso-occlusive crises. In this study, the failure of the 4 patients is probably due to the re-insults on the head of the femur as sequelae of vaso-occlusive crises. In conclusion, the use of ABMDO resulted in the improvement of the VAS, MHHP, and ASS and in desirable changes in the head of the affected femur, as seen with MRI.

## Conclusions

In conclusion, the results of this study give credence to our earlier short follow-up results showing that osteoblast transplantation is a good approach in healing of the avascular lesions in SCD. Our study fits the criteria of a Phase II clinical trial, and we believe a larger study equivalent to Phase III numbers should be undertaken and include patients with not only SCD but also steroid-induced and idiopathic avascular necrosis.

## Data Availability

is available at dsr@iau.edu.sa.
